# Pancreatic β-cell Dysfunction and Diabetes

**DOI:** 10.14789/ejmj.JMJ25-0001-R

**Published:** 2025-05-09

**Authors:** HITOSHI IIDA, HIROTAKA WATADA

**Affiliations:** 1 Department of Metabolism & Endocrinology, Juntendo University Graduate School of Medicine, Tokyo, Japan; 1 Department of Metabolism & Endocrinology, Juntendo University Graduate School of Medicine, Tokyo, Japan

**Keywords:** diabetes, insulin, pancreatic β-cells

## Abstract

The prevalence of diabetes continues to increase globally, posing a pressing issue for healthcare. Diabetes is primarily characterized by hyperglycemia, resulting from an absolute or relative deficiency in insulin activity, and is associated with systemic micro- and macro-vascular complications. Although diabetes encompasses multiple pathophysiological conditions based on its underlying mechanisms, pancreatic β-cell dysfunction is a common feature.

Pancreatic β-cells play a critical role in maintaining normal blood glucose levels by producing and secreting insulin in response to blood glucose concentrations. However, when β-cell dysfunction occurs, the cells fail to supply sufficient insulin to meet peripheral insulin demands, resulting in elevated blood glucose levels. Although multiple mechanisms contribute to β-cell impairment, these mechanisms often share overlapping pathways and can interact to exacerbate their detrimental effects.

Understanding the causes of β-cell dysfunction is essential for the prevention and treatment of diabetes. This review highlights the primary functions of pancreatic β-cells, including insulin biosynthesis and secretion, and provides an overview of the molecular mechanisms underlying β-cell dysfunction.

## Introduction

The global prevalence of diabetes is increasing, with an estimated 537 million people affected in 2021, projected to reach 783 million by 2045^1)^. Modern lifestyles, marked by high caloric intake and low physical activity, have significantly increased type 2 diabetes (T2D) cases, often linked to obesity^2)^. Diabetes, a metabolic disorder characterized by hyperglycemia, is associated with microvascular complications, such as retinopathy, nephropathy, and neuropathy, and a 2-to 4-fold higher risk of cardiovascular diseases^3, 4)^. These complications severely diminish patients' quality of life (QOL) and life expectancy.

Insulin, the only hormone reducing blood glucose levels, is secreted by pancreatic β-cells. Impaired insulin action leads to hyperglycemia, a major factor in diabetes mellitus. Diabetes management focuses on controlling blood glucose, body weight, blood pressure, and serum lipid profiles to prevent complications and ensure that patients maintain a quality of life similar to that of healthy individuals. Comprehensive knowledge of pancreatic β-cell function and diabetes pathogenesis is crucial for its prevention and treatment. This review aims to enhance the understanding of insulin action, its biosynthesis and secretion, and pancreatic β-cell dysfunction contributing to diabetes.

## Glucose metabolism and insulin action

Insulin regulates blood glucose levels primarily by suppressing hepatic glucose production and increasing glucose uptake in muscle and adipose tissue through its hormonal actions. Chronic overnutrition and physical inactivity can cause insulin resistance due to metabolic abnormal obesity. Pancreatic β-cells compensate it by hypersecreting insulin, but prolonged exposure of insulin resistance state leads to β-cell dysfunction and hyperglycemia^5)^. Most patients with T2D in Western populations are obese, with 89% having a BMI ≥ 25 kg/m^2^^6)^, while Japanese patients have an average BMI < 25 kg/m^2^^7)^. Asians have higher insulin sensitivity but lower insulin secretion than Caucasians^8)^, predisposing them to T2D at lower obesity levels.

As obesity and insulin resistance progress, insulin demand increases and β-cell function declines, leading to reduced insulin supply and rising blood glucose levels. Thus, understanding the mechanisms of pancreatic β-cell dysfunction is crucial for early diabetes detection and intervention.

## Physiology of pancreatic β-cells

### Structure of the islets of Langerhans

The pancreas maintains digestive and metabolic balance by secreting various enzymes and hormones. In adult mammals, it consists of exocrine and endocrine cells. Exocrine cells, primarily composed of acinar cells, produce digestive enzymes, while endocrine cells form the islets of Langerhans, which regulate glucose homeostasis through hormone secretion.

The human pancreas has about one million islets, with hormone-producing cells classified into five types:

• α-Cells secrete glucagon.

• β-Cells secrete insulin and amylin.

• δ-Cells secrete somatostatin.

• γ-Cells secrete pancreatic polypeptide.

• ε-Cells secrete ghrelin, rare.

These hormones are crucial for metabolic regulation and homeostasis^9)^. β-cells, which constitute 65-80% of islet cells, exclusively synthesize insulin. “β-cell mass” denotes the total β-cells in the pancreas, approximately 1 g in humans, predominantly located in the pancreatic tail, making in vivo visualization and quantification difficult. Current β- cell mass assessments rely on pathological analyses of pancreatic tissues from surgery or autopsy^10, 11)^. A study comparing β-cell mass in obese, non-diabetic Japanese individuals (mean BMI 28.5 kg/m^2^) and non-obese individuals (mean BMI 20.4 kg/m^2^) found no significant increase in β-cell mass in Japanese individuals^12)^. In contrast, studies on Caucasians showed significant increase in β-cell mass with obesity^13)^. This suggests that β-cell regeneration capacity in Japanese individuals may be lower than in Caucasians, influenced by genetic and environmental factors, contributing to the lower BMI manifestation of T2D in Asians compared to Western populations^14)^. Additionally, pathological studies indicate a significant reduction in β-cell mass in patients with T2D. Butler et al. found β-cell mass in lean patients with T2D reduced by about 40%, and in obese patients with T2D, by about 65% compared to non-diabetic controls matched for age and BMI^15)^. These findings highlight the crucial role of β-cell mass in T2D pathophysiology and the importance of understanding β-cell dynamics in the progression of diabetes.

### Insulin biosynthesis

Insulin biosynthesis begins with the expression of the insulin gene, which comprises three exons and two introns. The promoter region contains A, C, E, Z, and CRE elements crucial for β-cell- specific localization and serve as binding sites for pancreatic β-cell transcription factors, thereby regulating gene expression. These binding sites are within the -400 bp upstream of the transcription start site. Key transcriptional regulators such as PDX1, BETA2/NeuroD, and MafA drive the expression of preproinsulin mRNA, which ribosomes subsequently translate^16)^.

Preproinsulin mRNA, with a long half-life, becomes 2-3 times more stable in glucose-stimulated β-cells than in non-stimulated conditions. Its expression is acutely regulated by drugs, hormones, nutrients, particularly glucose, and its metabolites. This enhanced mRNA stability and cap-independent translation enable β-cells to meet sudden insulin demands during postprandial hyperglycemia^17)^.

Once translocated into the endoplasmic reticulum (ER) lumen, preproinsulin's signal peptide is cleaved, and it undergoes oxidative folding and disulfide bond formation, facilitated by protein disulfide isomerase (PDI) family members, with ERO1α/β regenerating PDI. Ca^2^^＋^-dependent chaperones like BiP assist in proinsulin folding. Misfolded proinsulin is identified and removed by ER-associated degradation (ERAD) and ER-phagy^18)^.

Proinsulin and processing enzymes are transported from the ER to the Golgi apparatus via COPII-mediated vesicular transport. In Golgi, proinsulin is packaged into secretory granules, with chromogranin A and B supporting protein condensation within immature granules. These granules bud from the Golgi and mature through microtubule- dependent processes, involving granule ion regulation, secretory responsiveness acquisition, proinsulin processing, protein condensation, and granule membrane stabilization^19)^.

Proinsulin conversion to mature insulin occurs in two steps. C-peptide cleavage at the B and A chain junctions, primarily involving PC1/3 in human β-cells and PC1/3 and PC2 in rodent β-cells. For this process, granule acidification and high Ca^2^^＋^ concentrations are essential. Carboxypeptidase E (CPE), requiring Zn^2^^＋^, further processes insulin. The mature insulin forms Zn^2^^＋^-centered hexamers, stored in the form of dense-core granules for release^20)^.

### Insulin secretion

Pancreatic β-cells possess a glucose-sensing mechanism that dynamically regulates insulin secretion to maintain blood glucose levels within the normal range. The rate of glucose metabolism is a critical determinant of the magnitude of the insulin secretory response as insulin secretion is regulated by ATP production and other metabolic coupling factors. Glucose is transported into β-cells via glucose transporters and is subsequently phosphorylated to glucose-6-phosphate by glucokinase (GK), which acts as the rate-limiting enzyme of glycolysis. This reaction allows GK activity to respond to changes in the physiological level of glucose concentration, thereby modulating glucose metabolism and insulin secretion. Glucose is eventually metabolized to pyruvate, which enters the mitochondrial tricarboxylic acid (TCA) cycle and promotes ATP production. An increase in ATP triggers the closure of ATP- sensitive potassium (K_ATP_) channels, leading to membrane depolarization, which opens voltage- dependent Ca^2^^＋^ channels. The resulting influx of Ca^2^^＋^ into the cytosol triggers exocytosis of insulin granules^21)^.

The primary physiological stimulus for insulin secretion is the postprandial increase in circulating glucose levels. Glucose-induced insulin secretion is mediated through two main pathways, ‘‘triggering’’ and ‘‘amplifying.” The triggering pathway involves the rapid rise in intracellular Ca^2^^＋^ concentration due to ATP production during glucose metabolism, which induces the exocytosis of insulin granules. This pathway is responsible for the first phase of insulin secretion, which is initially rapid and sharp. The amplifying pathway, which follows the triggering pathway, controls the second phase of insulin secretion, which is prolonged insulin release over several hours postprandially. This pathway is activated through K_ATP_ channel-independent mechanisms with various intracellular metabolic pathways that modulate this second phase. The insulinotropic effects of GLP-1 receptor agonists enhance insulin secretion by promoting this second-phase response.

In various types of diabetes, the impairment of these normal functions is a common pathological feature. The following chapter provides an overview of the different types of diabetes.

## Types of diabetes mellitus

Diabetes mellitus manifests as a result of various etiologies and is broadly classified into four categories. Despite differing causes, all forms of diabetes share a common feature, pancreatic β-cell dysfunction. This dysfunction results in an insufficient insulin supply, leading to hyperglycemia and subsequent disease onset.

### Type 1 diabetes (T1D)

Type 1 diabetes (T1D) is caused by the autoimmune destruction of pancreatic β-cells, resulting in complete or near-complete insulin deficiency. This condition typically manifests in childhood or adolescence but can occur at any age^22)^. Its pathogenesis involves the presentation of autoantigens by antigen-presenting cells, leading to T-cell activation. Activated T-cells release pro-inflammatory cytokines that destroy β-cells, resulting in absolute insulin deficiency. Patients with T1D require lifelong insulin therapy.

### Type 2 diabetes (T2D)

T2D accounts for approximately 90-95% of diabetes cases worldwide. It is characterized by a combination of insulin resistance due to metabolic abnormal obesity and β-cell dysfunction, leading to relative insulin deficiency. Genetic and environmental factors contribute to its development, and while it predominantly occurs in adults, its incidence among younger populations is increasing^23)^. In patients with T2D, β-cell dysfunction is evident from the early stages of the disease and progressively worsens over time. According to the UK Prospective Diabetes Study (UKPDS), β-cell function in patients with T2D is already reduced by approximately 50% at diagnosis and declines by approximately 5% annually thereafter^24)^. These data suggest that β-cell dysfunction begins approximately a decade prior to the clinical onset of T2D. Cross-sectional studies in Japanese patients with T2D also reported a significant and sustained decline in β-cell function, as indicated by C-peptide levels^25, 26)^. Chronic exposure to hyperglycemia suppresses β-cell function and reduces insulin biosynthesis and secretion while promoting apoptosis. Similarly, prolonged exposure to free fatty acids induces β-cell dysfunction through multifaceted mechanisms. Together, these factors exacerbate β-cell dysfunction known as glucolipotoxicity^27)^.

### Other types of diabetes

Mutations in regions of the insulin gene involved in transcription and translation, or those responsible for disulfide bond formation, can lead to the production of abnormal insulin, which exerts cytotoxic effects on pancreatic β-cells. These mutations impede proper insulin folding, retain misfolded variants in the ER, and induce ER stress and β-cell apoptosis. Additionally, genetic mutations in factors involved in β-cell differentiation, insulin biosynthesis, and insulin secretion can also lead to diabetes. These mechanisms underlie neonatal diabetes diagnosed within the first 12 months of life, mutant INS gene- induced diabetes of youth (MIDY), typically manifesting in childhood, and maturity-onset diabetes of the young (MODY), often developing during adolescence^28)^. MODY is classified into several types based on the causative gene. A genetic abnormality in GK, rate-limiting enzyme of glycolysis, causes MODY 2. Similarly, a genetic abnormality in pancreatic and duodenal homeobox 1 (PDX1), which plays a critical role in pancreatic islet development, is associated with MODY 4, while a genetic abnormality in neurogenic differentiation 1 (NeuroD1) is known to cause MODY 6. Many cases of MIDY and MODY require insulin therapy, however, MODY 1 and MODY 3, caused by mutations in the transcription factors hepatocyte nuclear factor 4α (HNF4A) and 1α (HNF1A), respectively, can often improve insulin secretion with sulfonylurea treatment. MODY 2 can sometimes achieve good blood glucose control through lifestyle modifications. Therefore, accurate diagnosis is essential^29)^.

Other types of diabetes include those associated with endocrine disorders, such as Cushing's syndrome, diabetes induced by hyperglycemia-causing drugs, such as steroids, and pancreatogenic diabetes due to pancreatic resection, as in cases of pancreatic cancer.

### Gestational diabetes mellitus (GDM)

Gestational diabetes mellitus (GDM) is commonly defined as hyperglycemia diagnosed or first occurring during pregnancy^30)^. In normal pregnancy, circulating levels of placental hormones, including human placental growth hormone (HPGH), corticotropin-releasing hormone (CRH), human placental lactogen (HPL), prolactin, estrogen, and progesterone, increase and induce insulin resistance. Among these, HPGH, which has a structure similar to pituitary growth hormone, plays a critical role in metabolic adaptations to ensure fetal nutrition. HPGH alters insulin receptor function through inhibiting PI3K activity and preventing the downstream of insulin signaling, which contributes to increased insulin resistance^31)^.

As maternal insulin resistance increases, postprandial glucose and free fatty acid levels increase, ensuring an adequate glucose supply for fetal growth. In normal pregnancies, maternal insulin secretion increases to maintain normoglycemia. Failure to adapt to these hormonal changes underlies GDM pathophysiology.

## Molecular mechanisms of pancreatic β-cell dysfunction

### Oxidative stress and mitochondrial dysfunction

Using glucose-derived metabolites, mitochondria produce ATP by consuming oxygen and produces reactive oxygen species (ROS) as byproducts. Oxidative stress occurs when ROS production surpasses the cellular antioxidant defense system, leading to β-cell apoptosis. Chronic hyperglycemia and elevated fatty acid levels alter mitochondrial number, inner membrane protein content, and morphology, releasing apoptotic factors. Long-chain fatty acids (FFA) also generate hydrogen peroxide (H_2_O_2_) via peroxisomal β-oxidation^32)^. Accumulated H_2_O_2_ induces oxidative stress in β- cells, causing mitochondrial DNA damage and mitochondrial fragmentation^33)^.

### Endoplasmic reticulum (ER) stress

Excessive proinsulin biosynthesis due to increased insulin demand overwhelms the ER's protein folding capacity, and mutations in proinsulin disrupt disulfide bond formation^34)^. Intracellular redox imbalances also hinder proper disulfide pairing. Cytokines and fatty acids reduce ER calcium levels, impairing chaperone functions, leading to misfolded proinsulin accumulation and impaired insulin synthesis and secretion^35)^. This is termed ER stress.

To counter ER stress, the unfolded protein response (UPR) is activated and mediated by IRE1, PERK, and ATF6^36)^.

• **PERK pathway**: Reduces ER protein load by phosphorylating eIF2α to suppress protein translation.

• **IRE1 pathway**: Activates endoribonuclease activity via autophosphorylation, causing unconventional splicing of XBP1 mRNA, upregulating ER chaperones, lipid biosynthesis enzymes, and ER-associated degradation (ERAD) genes.

• **ATF6 pathway**: Transports to the Golgi apparatus upon release from BiP, acting as a transcriptional activator for genes maintaining ER homeostasis and degrading unfolded proteins.

These pathways aim to restore ER homeostasis by reducing protein translation, enhancing folding capacity, and degrading misfolded proteins. However, failure of these mechanisms triggers cell death.

### Impaired autophagy

Autophagy is a highly conserved process in eukaryotic cells that maintains cellular homeostasis by degrading and recycling cellular components and damaged organelles^37)^. Accumulation of ubiquitinated proteins and damaged mitochondria has been observed in the pancreatic β-cells of autophagy-deficient mice^38, 39)^. In addition, impaired autophagy in pancreatic β-cells has been linked to increased vulnerability of β-cells to the cytotoxic effects of deposited amyloid, particularly human islet amyloid polypeptide (hIAPP), which contributes to the progression of β-cell failure^40)^. In fact, impaired autophagy has been observed in the β-cells of patients with T2D^41)^ and amyloid deposition in pancreatic islets is found in approximately 90% of these patients^42)^. High levels of free fatty acids and glucose further inhibit autophagic and lysosomal functions^43, 44)^, causing the development of T2D.

### Inflammation

In T1D, pancreatic β-cells are targeted and destroyed by islet antigen-specific T-cells via inflammatory cytokines, resulting in absolute insulin deficiency^45)^. Chronic risk factors for T2D, such as overweight, obesity, unhealthy diet, and physical inactivity, have been reported to induce inflammatory pathways over time^46)^. Inflammation is currently recognized as a common pathological condition in both T1D and T2D. Free fatty acids activate inflammatory pathways directly or indirectly via oxidative or ER stress. For example, palmitic acid induces the production of pro-inflammatory cytokines (IL-1β, IL-6, and IL-8), causing inflammatory cell damage^47, 48)^. Interactions between β-cells and immune cells promote the recruitment of M1 macrophages, further amplifying inflammatory responses^49)^.

### Interaction between stress pathways

**Figure 1:**
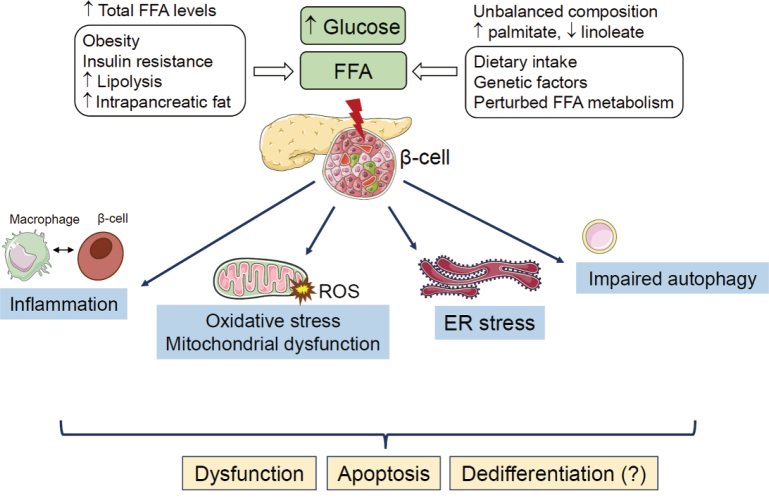
Molecular mechanisms of pancreatic β-cell dysfunction caused from glucolipotoxicity A prolonged elevation in free fatty acid (FFA) levels together with high glucose levels trigger stress responses in pancreatic β-cells. These responses include endoplasmic reticulum (ER) stress, oxidative stress marked by excessive reactive oxygen species (ROS) production, mitochondrial dysfunction, inflammation, and disruptions in autophagic processes. Interactions among these pathways can create feed-forward mechanisms, intensifying glucolipotoxic stress. Ultimately, these combined effects lead to β-cell dysfunction, cell death (apoptosis), and potentially a loss of their specialized function (dedifferentiation). (Modified quote from Ref. 27).

The multiple stress pathways described above act simultaneously or synergistically, forming a feed- forward mechanism that exacerbates glucolipotoxicity and β-cell dysfunction. ROS generation from oxidative stress depletes ER calcium stores, thereby triggering ER stress^50)^. ER stress amplifies inflammatory responses by inducing the expression of inflammatory genes^51)^. These mechanisms are summarized in Figure 1.

## Conclusion

This review summarizes current knowledge on β-cell dysfunction in diabetes. Evidence shows that loss of functional β-cell mass is central to all diabetes types. No therapies currently restore functional β-cell mass. Reducing β-cell burden is the most effective strategy for preserving β-cell mass, so diabetes management should focus on alleviating β-cell stress^52, 53)^. Effective treatment includes lifestyle modifications, especially dietary adjustments to limit calorie, carbohydrate, and fat intake, and regular physical activity to promote an active lifestyle^54, 55)^. Pharmacological interventions alone do not address the root causes of diabetes and may increase adverse events due to excessive medication reliance. Understanding β-cell dysfunction mechanisms and the impact of lifestyle changes on β-cell health can motivate patients to adopt proactive diabetes prevention and management strategies. Continued research is essential for developing novel therapies and improving outcomes for those at risk or living with diabetes.

## Funding

No funding was received.

## Author contributions

HI wrote the draft. HW reviewed the manuscript. All authors read and approved the manuscripts.

## Conflicts of interest statement

The authors declare that there are no conflicts of interest.
